# Autistic Traits Moderate the Impact of Reward Learning on Social Behaviour

**DOI:** 10.1002/aur.1523

**Published:** 2015-08-17

**Authors:** Maria Serena Panasiti, Ignazio Puzzo, Bhismadev Chakrabarti

**Affiliations:** ^1^ Centre for Integrative Neuroscience and Neurodynamics, School of Psychology & Clinical Language SciencesUniversity of Reading ReadingUnited Kingdom; ^2^ Department of PsychologySapienza University of Rome RomeItaly; ^3^ IRCCS, Fondazione Santa Lucia RomeItaly; ^4^ Department of PsychologyUniversity of SouthamptonSouthamptonUnited Kingdom

**Keywords:** reward, autism, prosocial behaviour, empathy

## Abstract

A deficit in empathy has been suggested to underlie social behavioural atypicalities in autism. A parallel theoretical account proposes that reduced social motivation (i.e., low responsivity to social rewards) can account for the said atypicalities. Recent evidence suggests that autistic traits modulate the link between reward and proxy metrics related to empathy. Using an evaluative conditioning paradigm to associate high and low rewards with faces, a previous study has shown that individuals high in autistic traits show reduced spontaneous facial mimicry of faces associated with high vs. low reward. This observation raises the possibility that autistic traits modulate the magnitude of evaluative conditioning. To test this, we investigated (a) if autistic traits could modulate the ability to implicitly associate a reward value to a social stimulus (reward learning/conditioning, using the Implicit Association Task, IAT); (b) if the learned association could modulate participants’ prosocial behaviour (i.e., social reciprocity, measured using the cyberball task); (c) if the strength of this modulation was influenced by autistic traits. In 43 neurotypical participants, we found that autistic traits moderated the relationship of social reward learning on prosocial behaviour but not reward learning itself. This evidence suggests that while autistic traits do not directly influence social reward learning, they modulate the relationship of social rewards with prosocial behaviour. ***Autism Res*** *2016, 9: 471–479*. © 2015 The Authors Autism Research published by Wiley Periodicals, Inc. on behalf of International Society for Autism Research

## Introduction

The social motivation hypothesis posits that atypical social behaviour in autism spectrum condition (ASC) could be caused by a failure to assign reward values to social stimuli and interactions [Chevallier, Kohls, Troiani, Brodkin, & Schultz, 2012; Dawson et al., 2004; Dawson, Webb, & McPartland, 2005]. Several studies have reported an aberrant functioning of the brain's reward circuit in individuals with high autistic traits and those with a clinical diagnosis of ASC. In some of these experiments, a reduced response to social rewards in comparison to nonsocial rewards was noted [Cox et al., 2015; Gossen et al., 2014; Schmitz et al., 2008; Scott‐van Zeeland, Dapretto, Ghahremani, Poldrack, & Bookheimer, 2010]. How such atypical ascription of reward value to social stimuli can lead to deficits in processes related to empathy has been demonstrated in a series of recent studies. Sims, Van Reekum, Johnstone, and Chakrabarti [2012] reported that spontaneous facial mimicry towards happy faces was enhanced by reward conditioning and that this enhancement was inversely related to individual autistic traits, i.e., the higher the autistic traits, the lower the reward‐driven enhancement of spontaneous facial mimicry. In a similar experiment using evaluative conditioning of hand stimuli, individuals with high autistic traits were found to engage in less automatic mimicry for hands associated with high vs. low rewards [Haffey, Press, O'Connell, & Chakrabarti, 2013]. Finally, autistic traits have been shown to modulate the connectivity between ventral striatum an inferior frontal gyrus in response to high vs. low conditioned happy faces [Sims, Neufeld, Johnstone, & Chakrabarti, 2014]. To summarise, these studies have demonstrated how autistic traits modulate a connection between the reward value of social stimuli and the extent of spontaneous/automatic mimicry they elicit.

However, a number of studies have also observed atypical reward related response to nonsocial reward (i.e., money) as well in ASC [Dichter et al., 2012; Kohls et al., 2013]. Thus, it remains unclear whether the observations reported above are due to impaired reward learning itself, or to an impaired link between the reward and empathy‐related processes.

The purpose of this study was to disentangle these two possibilities. With this aim, we used an evaluative conditioning paradigm [adapted from Sims et al., 2012] to associate the faces of two actors with two different levels of reward. Subsequently, we tested whether this conditioning affected the participants’ prosocial behaviour during a virtual ball tossing game (Cyberball Task, CT, [Williams, Cheung, & Choi, 2000]**)**. The cyberball task has been used widely to measure social behaviour, primarily to test the impact of social exclusion. In this study, we altered the paradigm to provide a proxy metric for prosocial behaviour (for a similar but distinct alteration, see [Riem, Bakermans‐Kranenburg, Huffmeijer, & van IJzendoorn, 2013]**).** Crucially, we used an Implicit Association Task (IAT) [Greenwald, Mcghee, Jordan, & Schwartz, 1998] to obtain a measure of the strength of conditioning. The IAT is typically used as a measure of automatically activated evaluations such as prejudices or stereotypes, but also as a measure of the association between the conditioned and unconditioned stimuli after classical conditioning protocols [Baccus, Baldwin, & Packer, 2004; Dijksterhuis, 2004; Hughes et al., 2005; Olson & Fazio, 2001]. We expected that the level of conditioning (measured by the IAT) would predict the strength of the prosocial behaviour in the CT.

Autistic traits are distributed continuously in the general population, where individuals with a clinical diagnosis of ASC are more represented at the high end of the score distribution. The aetiology of autistic symptoms has been shown to be comparable at extreme ends of the score distribution [Robinson et al., 2011]. Specifically for this study, we predicted that autistic traits might have a different influence on (a) the strength of conditioning (i.e., IAT) itself; and (b) the extent to which the conditioning was translated into prosocial behaviour.

## Participants

Fifty adults (26 females) between 18 and 41 years of age (*M* = 24.97; SD = 5.94) were recruited from the University of Reading campus area. All participants had normal or corrected‐to‐normal vision and six were left‐handed. None of the participants reported current neurological or psychiatric disorders, or history of regular drug/substance use. A total of seven participants were excluded from the analysis for: (a) technical problems (*n* = 4); (b) being outliers for the implicit association task (IAT) (*n* = 3). Thus, 43 participants were included in the analysis. All participants gave written informed consent and were financially remunerated for their participation. The study was approved by the School of Psychology and Clinical Language Sciences Research Ethics Committee of the University of Reading.

## Stimulus Materials

**Figure 1:**
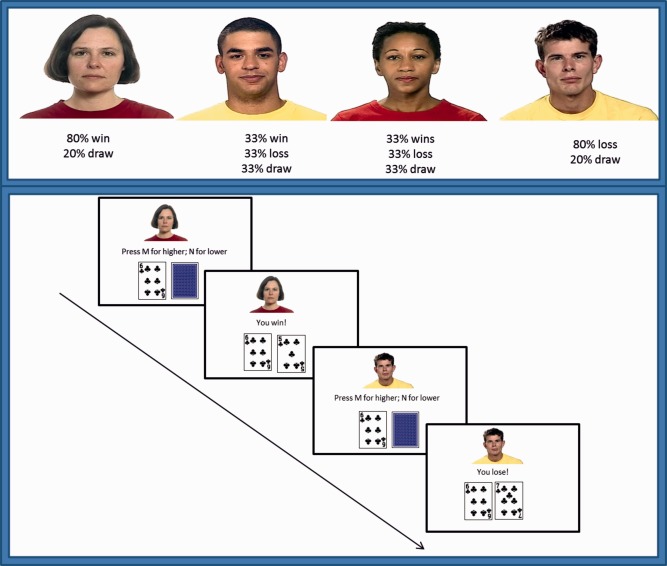
Top panel: example of the four neutral faces that were associated with different reward values (80% win, 33% win, 33% loss, 80% loss) during the conditioning phase. The first face corresponds to the Positive Reward condition (PosC), and the fourth face to the Negative Reward condition (NegC). Bottom panel: example of two trials of the condition phase in which the participants had to predict whether the face down card would be of lower or higher value than the face up card. Following their key response, feedback was displayed.

Stimuli in the conditioning phase consisted of static images of four faces (two males, two females) with neutral expressions (Figure 1). All stimuli were selected from the standardized Mindreading set [Baron‐Cohen, Golan, Wheelwright, & Hill, 2004; available at www.jkp.com/mindreading]. These stimuli show sufficient inter‐rater reliability and external validity [Golan & Baron‐Cohen, 2006; Golan, Baron‐Cohen, & Hill, 2006], and have been used in previous research [Sims et al., 2014, 2012].

## Procedure

Participants were seated in front of a computer screen and introduced to the experimental procedure. After the conditioning phase participants performed the Cyberball task and two versions of the IAT. The order of presentation of the two versions of the IAT was counterbalanced across participants. Importantly, the Cyberball task was always administered directly after the conditioning phase, in order to minimise extinction effects.

### Conditioning Phase

An evaluative conditioning paradigm in the form of a card guessing game was used to associate faces with high and low reward value (Figure 1). At the beginning of each trial, participants were shown two cards, one face up and one face down. The task was to predict whether the face‐down card was of greater or smaller value than the first card by pressing one of two keys on a keyboard. Participants knew that correct/incorrect predictions were associated with some monetary gain/loss (20 p). No money was won or lost if the cards were of equal value. After each response, an acoustic feedback indicating whether participants had won, lost or drawn the round was delivered for 1500 ms. The total amount of money won was shown after completion of the card game. In each trial, one of four emotionally neutral target faces was displayed on the top part of the screen with the cards below. The reward value associated with each face was manipulated by adjusting the number of trials in which participants won or lost money in the presence of this particular face. In the Positive Conditioned (PosC) condition, participants won 80% of the trials paired with the associated face; in the Negative Conditioned (NegC) condition, participants lost 80% of the trials in which the low rewarding face was presented. Two additional conditions in which participants won, lost or drawn the 33% of the trials, were used to prevent participants from detecting the underlying structure of the game. The remaining trials in all conditions were “draw” trials (i.e., the two cards were of the same value). The card game consisted of 120 randomized trials (30 trials per condition). The faces in the four conditions were counterbalanced across participants. To ensure that participants paid attention to the faces while playing the card game, they were told that during the test phase a simple memory task involving these same faces would be performed.

### Cyberball Game

**Figure 2:**
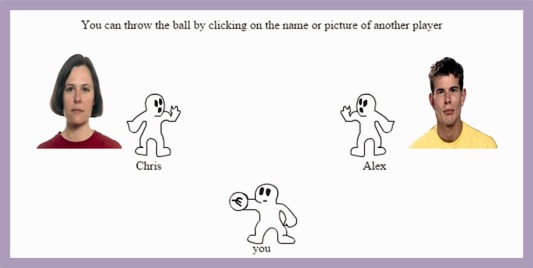
Screenshot of a Cyberball game trial. Participants were presented with a cartoon at the bottom of the screen. Pictures of the two fictional characters are displayed next to their respective cartoons. Participants had to decide whether to throw the ball to the PosC or the NegC face by clicking on the correspondent name.

After conditioning, participants were told that they needed to practise their mental visualization skills while playing a computer‐based ball tossing game with fictional characters. Participants were asked to click on the name of the player they wanted to throw the ball to and wait until the ball was tossed back to them. The ball‐tossing game lasted for 30 throws. The two fictional characters were the PosC and NegC faces (Figure 2). In order to rule out the possibility that the Cyberball task could itself influence the likeability of the faces, a reciprocation rate of 50% was programmed for each character meaning that it was equally likely that each character would toss the ball to the experimental participant or the other character. Participants were instructed not to pay attention to the tossing performance but to try and mentally visualize the experience to their best ability (e.g., where were they playing? what was the weather like?). The number of the throws to the PosC face and to the NegC face has been used as measures of induced prosocial behaviour. The performance on this task has already been shown to be correlated with social preference and prosocial behaviour [Andari et al., 2010; Riem et al., 2013].

**Figure 3:**
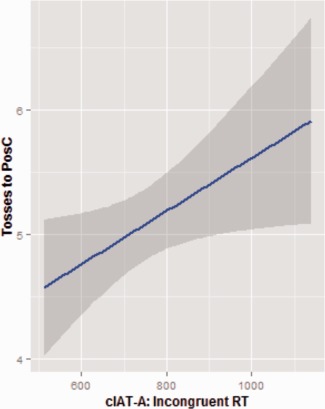
Correlation between RTs for the incongruent blocks of the cIAT‐A and the number of tosses directed towards the PosC face (*r* = 0.32, *P* = 0.041). The grey zone indicates 95% confidence intervals.

### Conditioning‐Implicit Association Task

The cIAT was used to test whether participants implicitly learned the association between wins and PosC face and between losses and NegC face. The cIAT consisted of a seven‐block design [Nosek, Greenwald, & Banaji, 2005] involving five practice blocks and two test blocks. Participants were instructed to use a right (P) or left (Q) key to categorize possible pictures belonging to one of the four categories (i.e., wins, losses, PosC identity or NegC identity) that appeared in the centre of the screen. In three blocks or 20 trials each (1, 2 and 5) participants had to respond according to two categories: either PosC vs. NegC identity or wins vs. losses. In the remaining blocks participants had to sort the pictures into four categories. Specifically, in two blocks [i.e., the congruent blocks: 3 (20 trials) and 4 (40 trials) for version cIAT‐A; 6 (20 trials) and 7 (40 trials) for version cIAT‐B] participants had to use one key to identify pictures that belonged either to the PosC identity *or* wins and another key to identify pictures of either NegC identity *or* losses. In the other two blocks [i.e., the incongruent blocks: 6 (20 trials) and 7 (40 trials) for version cIAT‐A; 3 (20 trials) and 4 (40 trials) for version cIAT‐B] participants had to use one key to identify pictures of PosC identity *or* losses and the other for NegC identity *or* wins. Each participant was administered two versions of the cIAT, one in which the congruent blocks appeared before the incongruent (cIAT‐A) and one with the opposite pattern (cIAT‐B). The sequence of administration of the two versions was counterbalanced across participants.

The stimuli consisted of the PosC and NegC pictures used during the conditioning paradigm, plus three pictures representing the concept of wins and three pictures representing the concept of loss (e.g., thumbs up and thumbs down, respectively).

### Trait Measures

Participants filled the Autism Spectrum Quotient (AQ) score online after they took part in the study. Four participants did not complete the questionnaire, thus, they were excluded from the AQ analysis; thus the total number of participants for these analyses was 39. Scores on the AQ ranged between 5 and 31 (*M* = 20.02 SD = 7.10). No participant scored above 32 on the full AQ, which has been found to be a reliable threshold score for a potential clinical diagnosis of ASC.

## Results

### cIAT

In order to control for the order of presentation, reaction times (RTs) of the cIAT were submitted to a 2 × 2 × 2 analysis of variance (ANOVA) with Congruency (congruent vs. incongruent) and Version (cIAT‐A vs. cIAT‐B) as within‐subject factors and Order of Presentation (A–B vs. B–A) as between‐subject factor. This ANOVA revealed a main effect of Congruency *F*(1,41) = 18.59, *P* < 0.001 η_p_^2^ = 0.31 showing that RTs for incongruent blocks were significantly higher than RTs for congruent blocks. The interaction between Congruency and Version showed to be significant *F*(1,41) = 55.62, *P* < 0.001 η_p_^2^ = 0.57 revealing that incongruent RTs were higher than congruent RTs only for the cIAT‐A version (*P* < 0.001, Tukey's corrected) (Table 1). The effect of the Order of presentation or its interactions were not significant (all *P*s > 0.05). Thus, irrespective of the order of presentation (A–B vs. B–A), the cIAT‐A version (where the congruent block was presented before the incongruent), was the only version that reflected the conditioning. Consequently, only the cIAT‐A version was used for the subsequent analysis.

**Table 1:** Means and Standard Errors for cIAT‐A and cIAT‐B Reaction Times

Version	Incongruent	Congruent
cIAT‐A	747.93 (23.87)	626.24 (14.24)
cIAT‐B	648.01 (18.32)	660.01 (15.79)

### Cyberball Task and c‐IAT

**Figure 4:**
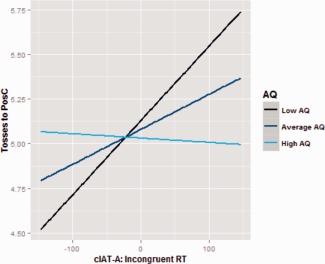
Autistic traits moderate the influence of conditioning (RTs for Incongruent trials of the cIAT‐A) on prosocial behaviour (Tosses to PosC). The learned association is transferred to a social preference only in individuals with low AQ.

There was no significant difference in the total number of balls tossed to the PosC identity vs. the NegC identity (*t*(42) = 0.87, *P* = 0.38). However, the number of balls tossed to the PosC identity significantly correlated with cIAT measure (Figure 3). Specifically, we found that slower RTs during the incongruent block of the cIAT‐A (i.e., a stronger implicitly learned association between wins and PosC face), were associated with greater number of balls tossed to the PosC identity (*r* = 0.32, *P* = 0.041) (Figure 4).

### Influence of autistic traits on implicit learning

To test whether autistic traits had an influence on implicit learning we used hierarchical mixed models. Data analysis was performed using R [R Development Core Team, 2013]. We performed a multilevel mixed linear regression analysis (LMM or “mixed effects models”; [Garson, 2013; Pinheiro & Bates, 2000], through the package lme4 ver. 1.1‐5 [Bates, Maechler, Bolker, & Walker, 2014]. Unlike traditional statistical methods, LMM are suitable for analyzing hierarchical data structures and accounting for the nonindependence of observations with correlated error by separately treating the effects caused by the experimental manipulation (fixed effects) and the ones that are not (random effects) [Pinheiro & Bates, 2000].

We considered the subject as a random factor (i.e., the random intercept), and Congruency, AQ and Congruency*AQ as fixed effects of our design. Only the Congruency predictor showed to be significant (*P* = 0.002, see Table 2).

**Table 2:** cIAT Task Performance: Beta Values for the Regression Model

	Estimate	SE	*t* value	*P*
Intercept	647.48	16.73	38.70	0.000
Congruency	47.91	16.08	2.97	0.002
AQ	−1.20	2.26	−0.53	0.594
Congruency*AQ	−1.15	2.20	−0.52	0.600

### Moderation Analysis

To test whether AQ traits could moderate the extent to which the learned association of faces with rewards (i.e., Incongruent RTs of cIAT‐A) was transferred to prosocial behaviour (i.e., Tosses to PosC), moderated multiple regression analysis was used. This analysis allows to test if the effect of the independent variable (Conditioning: Incongruent RTs of cIAT‐A) on the dependent variable (Tosses to PosC) is moderated by a moderator variable (AQ). The interaction is tested by entering in the regression model the two predictor variables and the product of the two (the interaction term) [Aiken & West, 1991]. First of all, we checked for bivariate outliers and no influential cases could be detected in the analyses (all Cook's distances < 1).

The interaction term (AQ*Conditioning) was found to be significant, showing that the extent to which the conditioning affected the dependent variable (the social preference) was moderated by participants’ autistic traits *b* = −0.0003, *t* = −2.14, *P* = 0.038. To further understand this interaction we conducted a simple slope analysis for high, average and low values of the moderator. We found that when AQ traits were low there was a significant positive relationship between level of conditioning and social preference, *b* = 0.0042, 95% CI [0.0014, 0.0069], *t* = 3.06, *P* = 0.004; when the AQ were on average or high the relationship failed to reach the significance (respectively, *b* = −0.0020, 95% CI [0.0000, 0.0039], *t* = 1.99, *P* = 0.053; *b* = −0.0002, 95% CI [−0.0032, −0.0028], *t* = −0.16, *P* = 0.87). This results show that only when AQ traits are low the learned conditioning is transferred to a social behaviour (Figure 4).

### Confounder Analysis

To control for a possible confound due to the gender of participants, all analyses were rerun using gender as a covariate. All effects reported above remained unchanged.

## Discussion

The central aim of this study was to test if autistic traits influenced the extent of reward learning or/and the extent to which the learned association translated into prosocial behaviour. We found three key results. First, the strength of conditioning was correlated to the number of ball tosses directed to the PosC face. Evaluative conditioning has already been seen to influence different proxy metrics related to empathy such as spontaneous mimicry of happy faces [Sims et al., 2012], or human hands [Haffey et al., 2013], cortical motor simulation [Trilla Gros, Panasiti, & Chakrabarti, 2015] and frontostriatal connectivity [Sims et al., 2014]. Importantly, evaluative conditioning is thought to contribute to several important phenomena in social psychology like stigmatization, and ingroup favoritism effect [Walther, Nagengast, & Trasselli, 2005] which respectively determine the way we categorize the social world and the way we favour some individuals (in‐group members) with respect to others (out‐group members). Consistently, here we demonstrate that it also plays a role in prosocial behaviour.

Second, while there was no evidence for any influence of autistic traits on the reward learning performance, we found that autistic traits moderated the extent to which reward learning for social stimuli was transferred to prosocial behaviour toward those faces. The first of these results is consistent with previous reports that demonstrated comparable reward learning behaviour in adults with and without Asperger Syndrome [Johnson, Yechiam, Murphy, Queller, & Stout, 2006] and comparable fear learning in adolescents with ASC [Bernier, Dawson, Panagiotides, & Webb, 2005]. In contrast, there are reports suggesting impaired fear and reward learning in ASC [Dawson, Meltzoff, Osterling, & Rinaldi, 1998; Dawson, Osterling, Rinaldi, Carver, & McPartland, 2001; Solomon et al., 2014; Zalla, Sav, & Leboyer, 2009]. The heterogeneity of the ASC samples in terms of symptom severity, age, as well as the experimental paradigms used in the studies above makes it difficult to draw any generalized conclusion about learning in ASC. However, it has been suggested that people with autism might have difficulties in reward learning when the reward feedback is not highly predictable [Dawson et al., 2002]. Thus, it is possible that the reinforcement scheduling (80%) we used was high enough to allow the acquisition of the association irrespective of AQ traits. This possibility should be further explored in future studies.

Crucially, we found that autistic traits moderated the extent to which the learnt reward value of the face translated to prosocial behaviour. Specifically, only participants with low autistic traits showed to transform the learned association into prosocial behaviour. This result is in line with the previous findings that showed that autistic traits modulate frontostriatal connectivity [Sims et al., 2014], and mimicry [Sims et al., 2012] for positive conditioned happy faces or human hands [Haffey et al., 2013]. It should however be noted that the samples for these studies, including the current one, are drawn largely from and around the university. Future studies should test the generalisability of these results in general population samples with larger age ranges.

Interestingly, the effect of the evaluative conditioning paradigm was reflected in the cIAT‐A, but not in the cIAT‐B. It has already been documented [Greenwald et al., 1998; Nosek, Greenwald, & Banaji, 2005] that the IAT version in which the congruent block is performed before the incongruent (like the cIAT‐A in our study) shows stronger effects than the other (the correspondent to cIAT‐B in our study). Consistent with this, a functional magnetic resonance imaging (fMRI) study reported a stronger activation of the cingulate cortex in the congruent‐before‐incongruent version of the IAT with respect to the incongruent‐before‐congruent one [Chee, Sriram, Soon, & Lee, 2000] suggesting that the former version is the most sensitive to measure the implicit association. Notably, in our study the congruent‐before‐incongruent (cIAT‐A) version was always more sensitive than the cIAT‐B no matter which version was administrated first. Furthermore, the cIAT‐B version did not show a significant advantage of the incongruent association with respect to the congruent one (i.e., faster reaction time for the incongruent than the congruent) but only a lack of difference in RTs between the two task conditions. These observations are important to rule out the possibility of a confound due to the order of presentation of the two versions of the cIAT task. Many studies have presented participants with the IAT‐A version only in order to make sure that the individual variability in the performance was not driven by this congruent‐before‐incongruent effect [Asendorpf, Banse, & Mücke, 2002; Egloff & Schmukle, 2002; Perugini & Leone, 2009]. However, we chose to administer both versions of the IAT to each of our participants, to check if the order of these tasks (cIAT‐A and cIAT‐B) had a significant effect on the observed results. While we do not see any effect of order, we note that switching from the congruent to the incongruent association makes the task performance more difficult (i.e., the difference in reaction times between congruent and incongruent trials is higher). This is not surprising, since the congruent trials are consistent with the direction of the original evaluative conditioning effects of the card game. On the other hand, doing the incongruent trials first did not reverse the evaluative conditioning effect, but made the difference in reaction times between the two conditions statistically insignificant. Since the aim of using the cIAT in this study was to provide a proxy measure of the strength of conditioning, we used only the cIAT‐A version, in light of the studies mentioned above.

By introducing independent measures of reward conditioning (i.e., cIAT) and social behaviour (CT) this study is able to parse the role that autistic traits might have on: (a) implicitly learning the stimulus‐reward association; (b) transferring the acquired reward value to prosocial behaviour. Our result expand upon the previous findings that showed link between reward and behavioural and neural markers related to empathy [Haffey et al., 2013; Sims et al., 2014, 2012]. Two key insights emerge from this study that helps understand these previous results better. First, all the previous studies used a single measure to index both conditioning and social behaviour (e.g., mimicry). In this experiment, we disentangle the two processes and test which of these two processes is modulated by autistic traits. Second, we provided evidence that autistic traits moderated the effect of reward conditioning not only on implicit measures like mimicry [as shown by Sims et al. 2012], but also on more explicit prosocial behaviour as measured with the cyberball task. That autistic traits can influence the process of transferring an acquired reward value to prosocial behaviour is particularly relevant for current treatments (ABA, Applied Behaviour Analysis) and intervention programs for autism that use operant or classical conditioning principles to improve social behaviour. Currently only a subset of patients who undergo ABA therapy show a good outcome [Howlin & Magiati, 2009; Matson & Smith, 2008], suggesting that there are a large number of patients for whom a reward‐conditioning based intervention does not necessarily lead to improvement of social skills. It is possible that deficits in this link between reward learning and empathy‐related behaviour can impact the generalization of the associations acquired during therapy to the everyday life. Future studies should test this result directly in people with ASC and stratify subgroups of patients who may/may not benefit from classical conditioning based treatment regimes.
